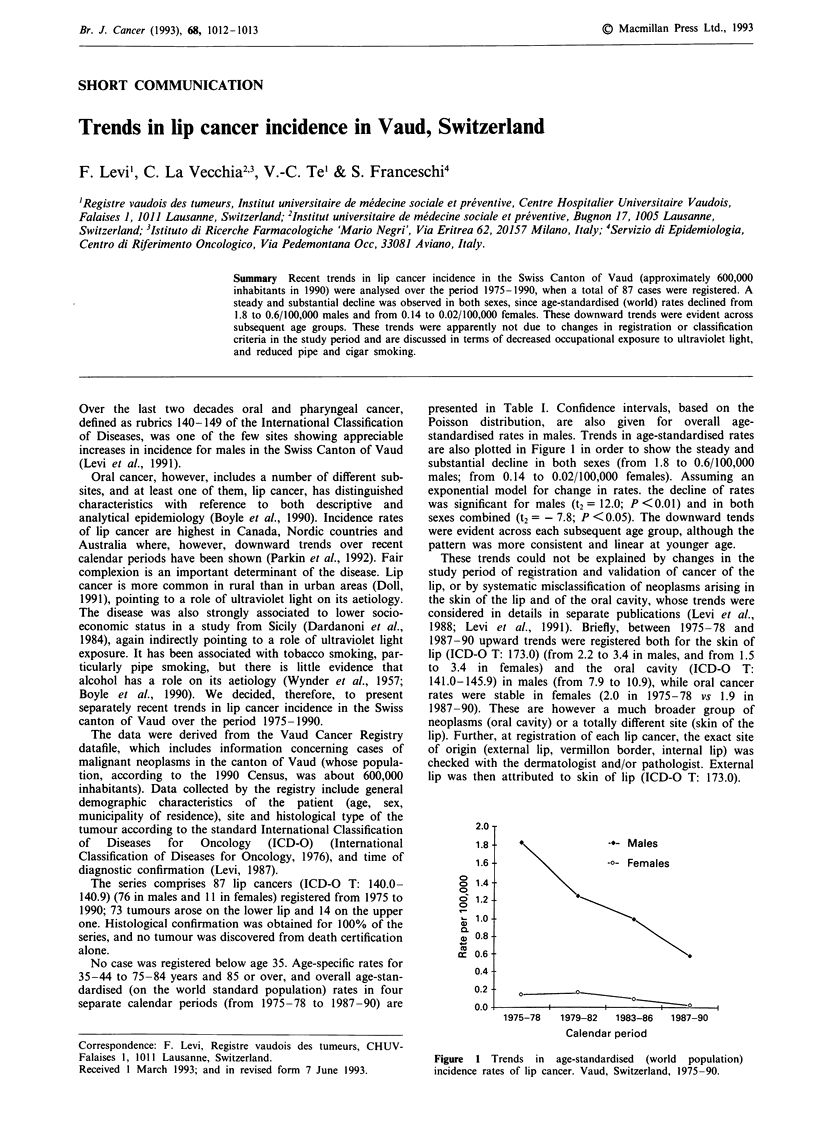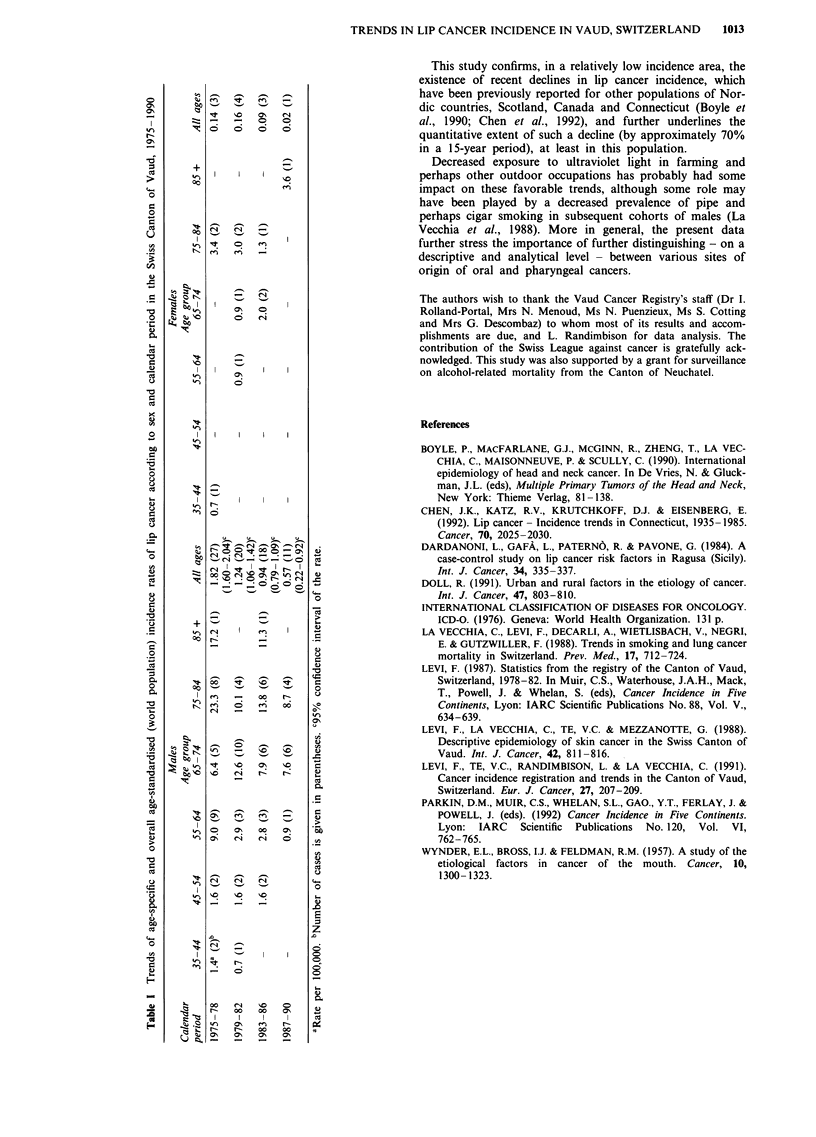# Trends in lip cancer incidence in Vaud, Switzerland.

**DOI:** 10.1038/bjc.1993.471

**Published:** 1993-11

**Authors:** F. Levi, C. La Vecchia, V. C. Te, S. Franceschi

**Affiliations:** Institut universitaire de médecine sociale et préventive, Centre Hospitalier Universitaire Vaudois, Lausanne, Switzerland.

## Abstract

Recent trends in lip cancer incidence in the Swiss Canton of Vaud (approximately 600,000 inhabitants in 1990) were analysed over the period 1975-1990, when a total of 87 cases were registered. A steady and substantial decline was observed in both sexes, since age-standardised (world) rates declined from 1.8 to 0.6/100,000 males and from 0.14 to 0.02/100,000 females. These downward trends were evident across subsequent age groups. These trends were apparently not due to changes in registration or classification criteria in the study period and are discussed in terms of decreased occupational exposure to ultraviolet light, and reduced pipe and cigar smoking.


*Br. J. Cancer* (1993), **68**, 1012–1013

© Macmillan Press Ltd., 1993

SHORT COMMUNICATION

### Trends in lip cancer incidence in Vaud, Switzerland

F. Levi[1], C. La Vecchia[2,3], V.-C. Te[1] & S. Franceschi[4]

[1]*Registre vaudois des tumeurs, Institut universitaire de médecine sociale et préventive, Centre Hospitalier Universitaire Vaudois, Falaises 1, 1011 Lausanne, Switzerland;* [2]*Institut universitaire de médecine sociale et préventive, Bugnon 17, 1005 Lausanne, Switzerland;* [3]*Istituto di Ricerche Farmacologiche 'Mario Negri', Via Eritrea 62, 20157 Milano, Italy;* [4]*Servizio di Epidemiologia, Centro di Riferimento Oncologico, Via Pedemontana Occ, 33081 Aviano, Italy.*

**Summary** Recent trends in lip cancer incidence in the Swiss Canton of Vaud (approximately 600,000 inhabitants in 1990) were analysed over the period 1975–1990, when a total of 87 cases were registered. A steady and substantial decline was observed in both sexes, since age-standardised (world) rates declined from 1.8 to 0.6/100,000 males and from 0.14 to 0.02/100,000 females. These downward trends were evident across subsequent age groups. These trends were apparently not due to changes in registration or classification criteria in the study period and are discussed in terms of decreased occupational exposure to ultraviolet light, and reduced pipe and cigar smoking.

Over the last two decades oral and pharyngeal cancer, defined as rubrics 140–149 of the International Classification of Diseases, was one of the few sites showing appreciable increases in incidence for males in the Swiss Canton of Vaud (Levi *et al.*, 1991).

Oral cancer, however, includes a number of different subsites, and at least one of them, lip cancer, has distinguished characteristics with reference to both descriptive and analytical epidemiology (Boyle *et al.*, 1990). Incidence rates of lip cancer are highest in Canada, Nordic countries and Australia where, however, downward trends over recent calendar periods have been shown (Parkin *et al.*, 1992). Fair complexion is an important determinant of the disease. Lip cancer is more common in rural than in urban areas (Doll, 1991), pointing to a role of ultraviolet light on its aetiology. The disease was also strongly associated to lower socio-economic status in a study from Sicily (Dardanoni *et al.*, 1984), again indirectly pointing to a role of ultraviolet light exposure. It has been associated with tobacco smoking, particularly pipe smoking, but there is little evidence that alcohol has a role on its aetiology (Wynder *et al.*, 1957; Boyle *et al.*, 1990). We decided, therefore, to present separately recent trends in lip cancer incidence in the Swiss canton of Vaud over the period 1975–1990.

The data were derived from the Vaud Cancer Registry datafile, which includes information concerning cases of malignant neoplasms in the canton of Vaud (whose population, according to the 1990 Census, was about 600,000 inhabitants). Data collected by the registry include general demographic characteristics of the patient (age, sex, municipality of residence), site and histological type of the tumour according to the standard International Classification of Diseases for Oncology (ICD-O) (International Classification of Diseases for Oncology, 1976), and time of diagnostic confirmation (Levi, 1987).

The series comprises 87 lip cancers (ICD-O T: 140.0–140.9) (76 in males and 11 in females) registered from 1975 to 1990; 73 tumours arose on the lower lip and 14 on the upper one. Histological confirmation was obtained for 100% of the series, and no tumour was discovered from death certification alone.

No case was registered below age 35. Age-specific rates for 35–44 to 75–84 years and 85 or over, and overall age-standardised (on the world standard population) rates in four separate calendar periods (from 1975–78 to 1987–90) are presented in Table I. Confidence intervals, based on the Poisson distribution, are also given for overall age-standardised rates in males. Trends in age-standardised rates are also plotted in Figure 1 in order to show the steady and substantial decline in both sexes (from 1.8 to 0.6/100,000 males; from 0.14 to 0.02/100,000 females). Assuming an exponential model for change in rates. the decline of rates was significant for males ($t_2 = 12.0$; $P < 0.01$) and in both sexes combined ($t_2 = -7.8$; $P < 0.05$). The downward tends were evident across each subsequent age group, although the pattern was more consistent and linear at younger age.

These trends could not be explained by changes in the study period of registration and validation of cancer of the lip, or by systematic misclassification of neoplasms arising in the skin of the lip and of the oral cavity, whose trends were considered in details in separate publications (Levi *et al.*, 1988; Levi *et al.*, 1991). Briefly, between 1975–78 and 1987–90 upward trends were registered both for the skin of lip (ICD-O T: 173.0) (from 2.2 to 3.4 in males, and from 1.5 to 3.4 in females) and the oral cavity (ICD-O T: 141.0–145.9) in males (from 7.9 to 10.9), while oral cancer rates were stable in females (2.0 in 1975–78 *vs* 1.9 in 1987–90). These are however a much broader group of neoplasms (oral cavity) or a totally different site (skin of the lip). Further, at registration of each lip cancer, the exact site of origin (external lip, vermillon border, internal lip) was checked with the dermatologist and/or pathologist. External lip was then attributed to skin of lip (ICD-O T: 173.0).

**Figure 1** Trends in age-standardised (world population) incidence rates of lip cancer. Vaud, Switzerland, 1975–90.

Correspondence: F. Levi, Registre vaudois des tumeurs, CHUV-Falaises 1, 1011 Lausanne, Switzerland.

Received 1 March 1993; and in revised form 7 June 1993.

This study confirms, in a relatively low incidence area, the existence of recent declines in lip cancer incidence, which have been previously reported for other populations of Nordic countries, Scotland, Canada and Connecticut (Boyle *et al.*, 1990; Chen *et al.*, 1992), and further underlines the quantitative extent of such a decline (by approximately 70% in a 15-year period), at least in this population.

Decreased exposure to ultraviolet light in farming and perhaps other outdoor occupations has probably had some impact on these favorable trends, although some role may have been played by a decreased prevalence of pipe and perhaps cigar smoking in subsequent cohorts of males (La Vecchia *et al.*, 1988). More in general, the present data further stress the importance of further distinguishing – on a descriptive and analytical level – between various sites of origin of oral and pharyngeal cancers.

The authors wish to thank the Vaud Cancer Registry's staff (Dr I. Rolland-Portal, Mrs N. Menoud, Ms N. Puenzieux, Ms S. Cotting and Mrs G. Descombaz) to whom most of its results and accomplishments are due, and L. Randimbison for data analysis. The contribution of the Swiss League against cancer is gratefully acknowledged. This study was also supported by a grant for surveillance on alcohol-related mortality from the Canton of Neuchatel.

### References

BOYLE, P., MACFARLANE, G.J., MCGINN, R., ZHENG, T., LA VECCHIA, C., MAISONNEUVE, P. & SCULLY, C. (1990). International epidemiology of head and neck cancer. In De Vries, N. & Gluckman, J.L. (eds), *Multiple Primary Tumors of the Head and Neck*, New York: Thieme Verlag, 81–138.

CHEN, J.K., KATZ, R.V., KRUTCHKOFF, D.J. & EISENBERG, E. (1992). Lip cancer – Incidence trends in Connecticut, 1935–1985. *Cancer*, **70**, 2025–2030.

DARDANONI, L., GAFÀ, L., PATERNÒ, R. & PAVONE, G. (1984). A case-control study on lip cancer risk factors in Ragusa (Sicily). *Int. J. Cancer*, **34**, 335–337.

DOLL, R. (1991). Urban and rural factors in the etiology of cancer. *Int. J. Cancer*, **47**, 803–810.

INTERNATIONAL CLASSIFICATION OF DISEASES FOR ONCOLOGY. ICD-O. (1976). Geneva: World Health Organization. 131 p.

LA VECCHIA, C., LEVI, F., DECARLI, A., WIETLISBACH, V., NEGRI, E. & GUTZWILLER, F. (1988). Trends in smoking and lung cancer mortality in Switzerland. *Prev. Med.*, **17**, 712–724.

LEVI, F. (1987). Statistics from the registry of the Canton of Vaud, Switzerland, 1978–82. In Muir, C.S., Waterhouse, J.A.H., Mack, T., Powell, J. & Whelan, S. (eds), *Cancer Incidence in Five Continents*, Lyon: IARC Scientific Publications No. 88, Vol. V., 634–639.

LEVI, F., LA VECCHIA, C., TE, V.C. & MEZZANOTTE, G. (1988). Descriptive epidemiology of skin cancer in the Swiss Canton of Vaud. *Int. J. Cancer*, **42**, 811–816.

LEVI, F., TE, V.C., RANDIMBISON, L. & LA VECCHIA, C. (1991). Cancer incidence registration and trends in the Canton of Vaud, Switzerland. *Eur. J. Cancer*, **27**, 207–209.

PARKIN, D.M., MUIR, C.S., WHELAN, S.L., GAO., Y.T., FERLAY, J. & POWELL, J. (eds). (1992) *Cancer Incidence in Five Continents*. Lyon: IARC Scientific Publications No. 120, Vol. VI, 762–765.

WYNDER, E.L., BROSS, I.J. & FELDMAN, R.M. (1957). A study of the etiological factors in cancer of the mouth. *Cancer*, **10**, 1300–1323.